# Tuning the physical properties of ultrathin transition-metal dichalcogenides *via* strain engineering

**DOI:** 10.1039/d0ra07288e

**Published:** 2020-10-27

**Authors:** Yalan Yan, Shuang Ding, Xiaonan Wu, Jian Zhu, Dengman Feng, Xiaodong Yang, Fangfei Li

**Affiliations:** Institute for Interdisciplinary Biomass Functional Materials Studies, Jilin Engineering Normal University No. 3050 Kaixuan Road Changchun 130052 People's Republic of China y86908051@126.com; State Key Laboratory of Superhard Materials, College of Physics, Jilin University No. 2699 Qianjin Street Changchun 130012 People's Republic of China lifangfei@jlu.edu.cn; Department of Chemical Engineering, Chengde Petroleum College Chengde 067000 People's Republic of China cdpcwxn2007@163.com

## Abstract

Transition-metal dichalcogenides (TMDs) have become one of the recent frontiers and focuses in two-dimensional (2D) materials fields thanks to their superior electronic, optical, and photoelectric properties. Triggered by the growing demand for developing nano-electronic devices, strain engineering of ultrathin TMDs has become a hot topic in the scientific community. In recent years, both theoretical and experimental research on the strain engineering of ultrathin TMDs have suggested new opportunities to achieve high-performance ultrathin TMDs based devices. However, recent reviews mainly focus on the experimental progress and the related theoretical research has long been ignored. In this review, we first outline the currently employed approaches for introducing strain in ultrathin TMDs, both their characteristics and advantages are explained in detail. Subsequently, the recent research progress in the modification of lattice and electronic structure, and physical properties of ultrathin TMDs under strain are systematically reviewed from both experimental and theoretical perspectives. Despite much work being done in this filed, reducing the distance of experimental progress from the theoretical prediction remains a great challenge in realizing wide applications of ultrathin TMDs in nano-electronic devices.

## Abstract 2

Transition-metal dichalcogenides (TMDs) have become one of the recent frontiers and focuses in two-dimensional (2D) materials fields thanks to their superior electronic, optical, and photoelectric properties.

## Graphical abstract

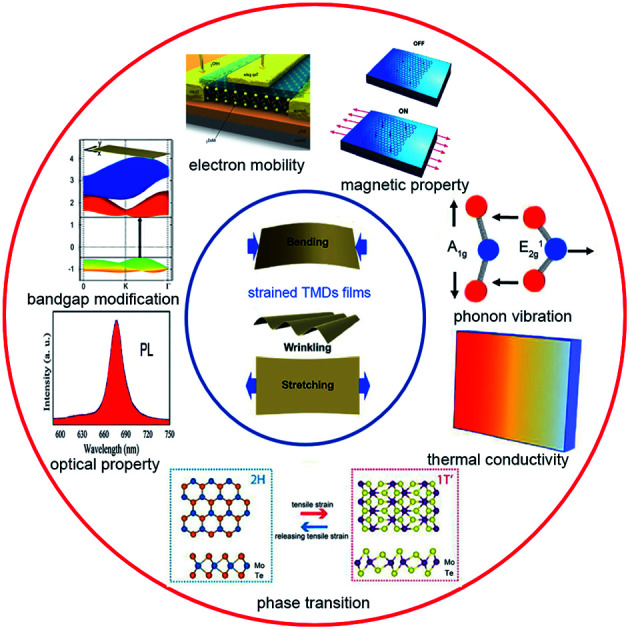

## Introduction

Since the successful exfoliation of graphene from graphite in 2004,^1^ 2D materials have attracted great attention in the scientific community thanks to their novel physical properties.^2–6^ Among all the 2D materials, TMDs with the formula MX_2_, where M is a transition mental element and X is a chalcogen, assume a central place in the research of layered materials, since these materials possess great potential in the fields of electronics, optics and optoelectronics,^7,8^ and they are chemically versatile and naturally abundant. TMDs usually crystalize in three types, namely 2H-MX_2_, 1T-MX_2_, and 3R-MX_2_, depending on the stacking configuration of X–M–X.^9^ In each X–M–X layer, the M–X bond is strongly covalent, while adjacent layers are weakly coupled by van der Waals (vdW) interactions, resulting in a distinctive easy slippage and easy cleavage of planes in TMDs.

At present, the synthesis of ultrathin TMDs has been widely investigated and many excellent review papers are available.^10–12^ However, these materials may not be directly useful for applications requiring properties other than their natal ones. Thus, it is the right time to think about not only synthesizing materials but also modifying their physical properties. Considering the fact that, ultrathin TMDs are intrinsically capable of sustaining much larger mechanical strain when compared to their bulk counterparts,^13–15^ meanwhile, for ultrathin TMDs based nanoscale devices, the TMDs films are usually under strain, thus, strain engineering should be a perfect approach to bring in novel and superior physical properties in ultrathin TMDs by tuning their lattice and electronic structure. In detail, strain can effectively change atomic bond-configuration (bond length, bond angle and bond strength) and the interaction between electronic orbitals, resulting in the emergence of novel phenomena and properties in ultrathin TMDs, such as thermal, electronic, optical, and magnetic properties. In this way, the capability to change the intrinsic properties of ultrathin TMDs through strain engineering has created tremendous opportunities for their applications in a variety of fields, including sensing, electronic and photoelectric devices, and so on.

At present, the study subjects of recent reviews on the strain engineering of ultrathin films are usually extended to 2D materials, including graphene, black phosphorus, BN, and TMDs, *etc.* For instance, Liu *et al.* summarized the current methods of introducing strain into 2D materials by classifying the deformation modes, and reviewed the crucial role of 2D material-substrate interfaces in governing these deformations.^16^ Sun *et al.* discussed the fundamentals of strain engineering in 2D materials from macro and atomic perspective and illuminated the effects of strain on the physical and chemical properties in 2D materials.^17^ Roldán *et al.* reviewed the recent progress in controlling the optical and electronics properties of 2D materials by strain engineering.^18^ Considering the fact that, the various 2D materials have their obviously different responses to strain, it should be mentioned that, narrowing the scope of study subjects to a smaller group will lead to a more systematic summary of strain engineering in ultrathin film, thus, a more systematic review on the strain engineering of ultrathin TMDs should be of great significance.

In this review, we focus on the recent development on the regulation of physical properties of ultrathin TMDs *via* strain engineering, we first discuss the characteristics of currently employed approaches for introducing strain by external force, subsequently, the recent advances on the strain engineering of ultrathin TMDs are reviewed by categorizing the physical properties, moreover, some of the bottle-neck problems in current researches are also pointed out.

## Strain engineering for ultrathin TMDs

### Approaches for introducing strain

Up to now, various experimental methods and strategies for generating strain have been implemented to explore novel physical properties in ultrathin TMDs, including externally exerted strain and internally evoked strain. External strain mainly involves external force, or stimulation, such as bending, stretching of flexible substrates and substrate thermal expansion. While internal strain mainly originates from multiple components and surface/interface interactions, which are closely related to defects, or lattice mismatches. Despite the diversity of experimental strategies for generating strain, an effectively controllable strain should be of great significant to obtain a targeted physical property, which is difficult to obtain by taking advantage of internal evoked strain owing to the unmanageable defects and lattice mismatches. Thus, in this review, we mainly focus on the research progress in the structures and properties of TMDs films under external strain.

**Fig. 1:**
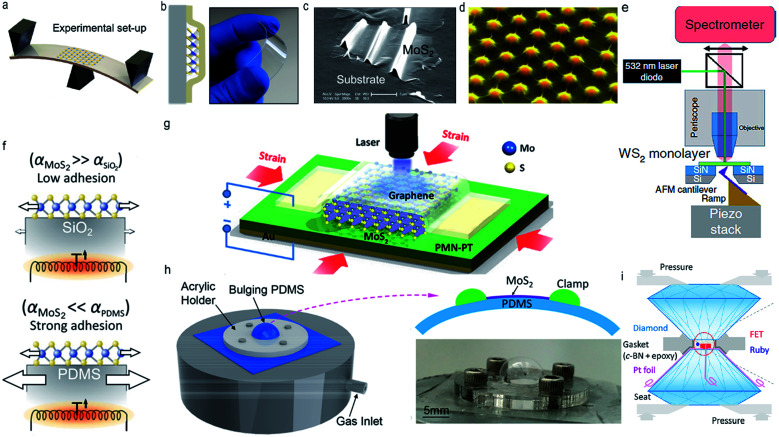
The schematic diagram of approaches for introducing external strain. (a) The three-point bending apparatus. Reproduced with permission.^19^ Copyright 2013, American Physical Society. (b) Large strain magnitude can be introduced in polymer encapsulated monolayer WSe_2_ by bending apparatus. Reproduced with permission.^20^ Copyright 2018, American Chemical Society. (c) Wrinkled monolayer MoS_2_ fabricated by prestretching an elastomeric substrate. Reproduced with permission.^21^ Copyright 2013, American Chemical Society. (d) Strained monolayer MoS_2_ indented by SiO_2_ nanocones, where the strain on the tips of nanocones exhibit highest while the strain between nanocones is lowest. Reproduced with permission.^22^ Copyright 2015, Nature. (e) Schematic of an atomic force microscope (AFM)-based apparatus. Reproduced with permission.^23^ Copyright 2020, Nature. (f) Schematic of the thermal-expansion induced strain in monolayer MoS_2_. Reproduced with permission.^27^ Copyright 2015, Institute of Physics. (g) Schematic diagram of optical measurements on strained trilayer MoS_2_ which is sandwiched between a piezoelectric substrate and a graphene electrode. Reproduced with permission.^28^ Copyright 2013, American Chemical Society. (h) Experimental setup for blown-bubble bulge measurement, high strain can be achieved in suspended MoS_2_ films by slowly diffusing N_2_ gas through the PDMS substrate to pressurize the cavity. Reproduced with permission.^29^ Copyright 2017, American Chemical Society. (i) Schematic of the electrical conductance measurements of few-layer MoS_2_ in a diamond anvil cells. Reproduced with permission.^30^ Copyright 2017, American Chemical Society.

In this section, we summarize the feasible and currently employed approaches for introducing external strain in ultrathin TMDs: bending a flexible substrate, polymer-based encapsulation, formation of wrinkles, patterned substrate, atomic force microscope (AFM)-based apparatus, substrate thermal expansion, piezoelectric stretching, and diamond anvil cells (DAC). The schematic diagrams of experimental approaches for generating external strain are presented in Fig. 1 as below.

According to the previous reports, by bending/streching a flexible substrate, a uniaxial tensile strain of up to 4% can be acquired in ultrathin TMDs,^19,31^ as long as the TMDs membranes can be easily transferred and attached to the hosting substrate (Fig. 1a).

Despite the universal application of this technique above, it also presents some serious shortcomings, such as the difficulties in verifying the applied strain and obtaining large strain magnitude. In view of the above-mentioned bottlenecks, a novel polymer-based encapsulation method was developed to obtain large range accurate, uniform, highly repeatable, and independently measurable strain in ultrathin TMDs. In detail, firstly, ultrathin MoS_2_ was lifted from the SiO_2_/Si substrate by using cellulose acetate butyrate, then it was encapsulated between two polycarbonate substrates which are tightly bonded together, in this way, strain can be introduced in ultrathin MoS_2_ by bending the polymer substrate^20^ (Fig. 1b).

In addition, strain can also be brought in by fabricating wrinkles in supported ultrathin TMDs. Firstly, the elastomeric substrate was prestretched, on which ultrathin MoS_2_ was deposited, subsequently, the prestretching strain in elastomeric substrate is suddenly released and wrinkles generate in loaded ultrathin TMDs due to the buckling-induced delamination. In this way, local strain is attained in TMDs film thanks to the large deformation in wrinkled zone^21^ (Fig. 1c). It is notable that the amplitude and periodicity of wrinkles in ultrathin TMDs depend largely on the prestreching strain in elastomeric substrate and the releasing rates of prestrain.

Owing to the flexible characters of ultrathin TMDs and their van der Waals interaction to the substrate, local deformation of TMDs films can be directly obtained by transferring TMDs films onto a patterned substrate. This strategy has led to the rapid development of pattern design of substrate, along with the studies on the strain-induced interface chemistry and physics in ultrathin TMDs. For example, Mangu *et al.* established a locally varying strain fields in 6 layer MoS_2_ through conformal contact of ultrathin MoS_2_ with a textured Si substrate, realizing the facilely modification of the electronic band structure of ultrathin MoS_2_.^32^ Zheng *et al.* designed a strained MoS_2_ film using a patterned nanocone substrate and discussed their application in the hydrogen evolution reaction and bandgap engineering^22,33,34^ (Fig. 1d).

In addition, an AFM-based setup is also an effective approach to introduce non-uniform mechanical strain in ultrathin TMDs by indenting the film with an AFM tip.^23–26^ As shown in Fig. 1e, the WS_2_ monolayer suspended on top of a hole in the substrate membrane is indented from the bottom by an AFM cantilever and being optically interrogated from the top, in this way, the dynamics of the excited carriers in strained monolayer WS_2_ can be revealed by optical spectroscopy.^23^

It is worth mentioning that approaches in Fig. 1a–c can only introduce a uniaxial strain, however, the effect of biaxial strain in modifying the physical properties of ultrathin TMDs is obviously stronger than that of uniaxial strain (a more detailed discussion is presented in Section 2.2), thus, experimental realization of biaxial strain is of great significant. At present, there are two main ways to introduce biaxial strain, namely substrate thermal expansion and piezoelectricity. As shown in Fig. 1f, biaxial strain can be applied in supported TMDs films by heating the sample using a focused laser beam, profiting from the mismatch of the thermal expansion between ultrathin films and the substrate.^27^ Both tensile and compressive strain can be obtained depending on the coefficient of thermal expansion of substrate and ultrathin TMDs. In addition, by using piezoelectric actuators, biaxial compressive strain up to 0.2% can be obtained in a TMDs film supported by a piezoelectric substrate and covered by a transparent graphene electrode^28^ (Fig. 1g).

Except from the uniaxial and biaxial strain, isotropic strain can also be introduced by fabricating bubble/tents in ultrathin TMDs. In detail, many applications of 2D materials films involve multiple transfer processes of 2D materials to a substrate, bubbles/tents can be formed frequently by trapping water, gas, or solid nanoparticles at the interface of 2D materials,^35–39^ in this way, considerable in-plane strain associated with these out-of-plane bubbles/tents can be successfully introduced in ultrathin films, and their electronic structure and optical properties can also be tuned effectively. For instance, according to the report by Yang *et al.*, isotropic strain up to 3.5% can be acquired in ultrathin MoS_2_ on a flexible substrate by using “blown-bubble” bulge technique, (Fig. 1h) opening a new pathway to continuously modify the optical signatures of MoS_2_ films.^29^ Sun *et al.* reported a simultaneous generation of three PL peaks in multilayer MoS_2_ bubbles, which can be attributed to the strain-induced weakened interlayer coupling in multilayer MoS_2_ bubbles.^40^ In addition, bubbles can also be created during the fabrication of a van der Waals (vdW) heterostructures. In detail, contamination such as adsorbed water and hydrocarbons inevitably exist on the surfaces of assembled crystals, during the assembly, vdW forces attract the crystals together, naturally, contamination was squeezed out and trapped into sub-micrometer-size bubbles.^41^

Apart from the in-plane strain, an out-of-plane strain with the magnitude of GPa can also be introduced in ultrathin TMDs by implementing DAC technique (Fig. 1i). The pressure is usually monitored using the ruby fluorescence method, and a hydrostatic/uniaxial compression can be obtained in ultrathin TMDs by adding/not involving pressure transmitting medium in the pressure chamber of DAC.^30,42^

### Tuning physical properties of ultrathin TMDs *via* strain engineering

In recent years, with the rapid development of exfoliation and transfer technique of nanomembranes, tremendous progresses have been made in the investigations of strained ultrathin TMDs. In the following subsections, we summarize the recent development in the strain engineered physical properties (lattice structure, phonon vibration, phase transition, thermal transport property, electronic structure, photoluminescent property, electrical transport property, and magnetic property) of ultrathin TMDs, which may provide necessary guidance and direction for the applications of ultrathin TMDs in electronics, optoelectronics, catalysis, and so on.

#### Lattice structure, phonon properties, phase transition, and thermal transport properties

**Fig. 2:**
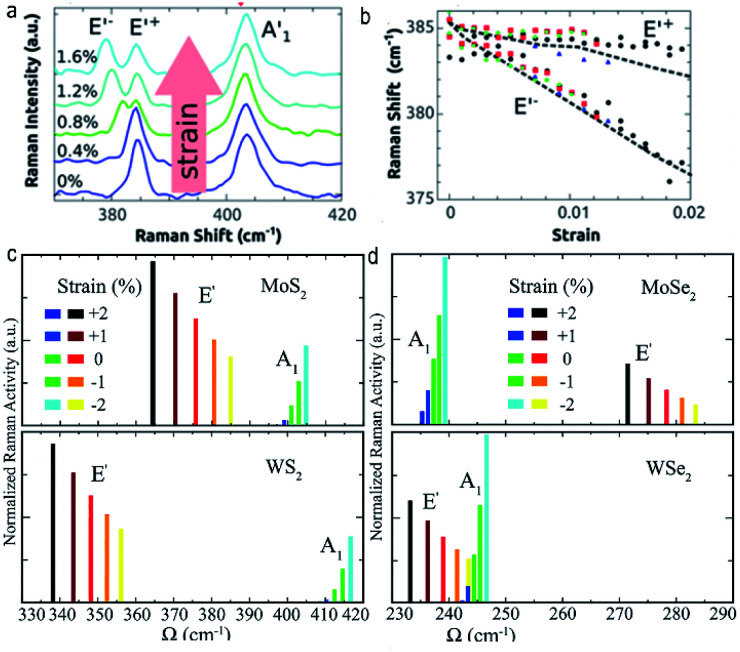
(a) Raman spectra of monolayer MoS_2_ under different strain. (b) Strain-dependent Raman frequency of mode E′ in monolayer MoS_2_. Reproduced with permission.^50^ Copyright 2013, American Chemical Society. The calculated strain responses of Raman activities of monolayer (c) MoS_2_ and WS_2_ and (d) MoSe_2_ and WSe_2_. Reproduced with permission.^53^ Copyright 2018, American Physical Society.

Generally speaking, strain can lead to the changes of bond length and angle, namely lattice deformation,^43–45^ this will in turn alter the phonon properties of ultrathin TMDs, which can be detected by Raman spectroscopy. For instance, both experimental observations and first-principles calculations have confirmed the blue/red shift of in-plane vibrational E^1^_2g_ mode in ultrathin group-VI TMDs under compressive/tensile strain.^28,46,47^ Moreover, the splitting of E^1^_2g_ mode was also observed in ultrathin group-VI TMDs upon uniaxial strain,^19,31,48–52^ whereas, compared to the splitting E^1^_2g_ mode, the A_1g_ mode is more inert to the in-plane strain thanks to its out-of-plane vibration characteristic,^20,48,53^ (Fig. 2a and b) indicating that the vibrational responses of Raman modes should be directly related to the relative orientation between the vibrational direction and strain direction. Meanwhile, not only the Raman frequencies, but also Raman activities of ultrathin TMDs will undergo a prominent change under tensile strain, *i.e.*, previous *ab initio* calculations have demonstrated that the application of tensile strain can induce increased (decreased) activity of E′ (A_1_) in group-VI TMDs (MX_2_, M = Mo, W; X = S, Se), while the application of compressive strain will lead to an opposite responses of Raman activity between E′ and A_1_ modes, indicating that the activity ratio of the E′ to A_1_ phonon mode is a key to determine the strain type^53^ (Fig. 2c and d).

Sometimes, strain-induced phase transition can also be observed in strained ultrathin TMDs. For example, both experimental observations and theoretical calculations have demonstrated a phase transition of monolayer MoTe_2_ from semiconducting 2H phase to metal 1T′ phase, once a tensile strain of 0.3% is applied in it,^54,55^ indicating potential applications of monolayer MoTe_2_ in electronic and nanoelectromechanical devices.

In addition, it is known that the heat conduction process of semi-conductive and insulated materials are mainly dependent on phonons, electrons contribute very little even at low temperature, thus, strain engineering is an effective mean to modify the thermal conductivity of semi-conductive ultrathin TMDs owing to its softening/stiffening of phonon modes upon tensile/compressive strain.^56^ A low lattice thermal conductivity is favored in thermoelectric^57,58^ and thermal rectification^59^ applications, while a high lattice thermal conductivity is preferred in power sources and transistors to dissipate waste heat efficiently.^60^ However, the influences of strain on the thermal transport properties of ultrathin 2D materials are unpredictable, since the completely opposite responses of out-of-plane acoustic (ZA) mode with the transverse acoustic (TA) and longitudinal acoustic (LA) modes upon tensile/compressive strain. In detail, upon tensile strain, the ZA mode of two-dimensional (2D) materials becomes harder whereas the TA and LA modes become softened.^61^ Thus, the lattice thermal conductivity of ultrathin TMDs should be directly related to the contribution percentage of ZA, TA and LA. For instance, according to the first-principles calculations and the Boltzmann transport equation (BTE), at ambient condition, for both monolayer 2H-MoTe_2_ and monolayer ZrTe_2_, the rotational symmetry of the out-of-plane mode causes a quadratic ZA modes near the high symmetric *Γ* point, and ZA mode is the main contributor of the lattice thermal conductivity.^61,62^ Whereas, as tensile strain increases, the rotational symmetry is broken, the ZA mode changes from a quadratic nature to a linear one, resulting in a decrease (increase) in the contribution from the ZA (TA/LA) mode, thus, the lattice thermal conductivity of monolayer exhibit a dramatically reduction upon tensile strain.^61,62^

At present, the studies on the thermal transport properties of strained ultrathin TMDs still remain on the level of theoretical calculations. The relative experiment investigations are difficult to implement due to the following reasons: (1) the large specific surface area of ultrathin TMDs should lead to a large thermal radiation, which will undoubtedly influence the experimental results of thermal conduction. (2) The introduction of strain in ultrathin TMDs is totally dependent on the substrate, the close contact between substrate and TMDs films and the intrinsic ultrathin character of TMDs film make it difficult to calibrate the temperature of TMDs films, leading to a stalling state in experimental measurements, more work should be done to obtain breakthrough progress in experimental studies.

#### Electronic, optical and electrical transport properties

**Fig. 3:**
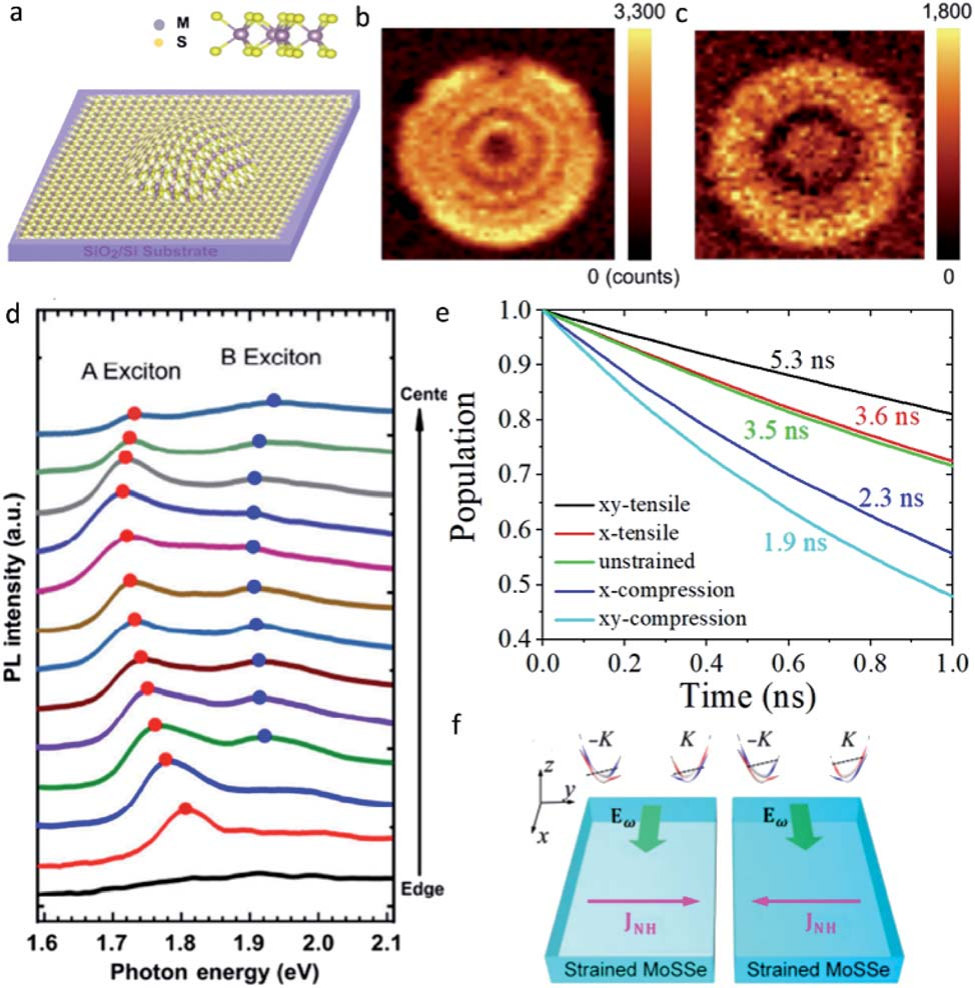
(a) Schematic diagram of a MoS_2_ bubble with isotropic tensile strain. PL intensity mapping images of strained MoS_2_ bubble at the resonant energies of (b) A excitons and (c) B excitons. (d) PL spectra of four-layer MoS_2_ as a function of radial coordinate over MoS_2_ bubble, whose center is defined as *r* = 0. Reproduced with permission.^67^ Copyright 2020, Springer. (e) Nonradiative exciton recombination dynamics of monolayer WSe_2_ at different strains. Reproduced with permission.^68^ Copyright 2019, American Chemical Society. (f) Schematic diagram of strong gate dependence of nonlinear Hall effects in strained MoSSe. Reproduced with permission.^69^ Copyright 2020, American Physical Society.

Different from the almost unaffected electronic properties of graphene under strain, the electronic properties of ultrathin TMDs are significantly sensitive to almost all types of mechanical strain, namely shear strain, tensile strain and compressive strain, the strain-induced electronic structural evolution will undoubtedly result in the modification of optical and electrical transport properties.^63–66^ For instance, the existence of continuously varying local strain on few-layer MoS_2_ bubbles can lead to an increasing PL intensity from the center of the bubble to the edge due to the strain-induced indirect to direct bandgap transition, which greatly extends the application of these materials in optoelectronic devices^67^ (Fig. 3a–d). Within 1% strain range, a varying charge carrier lifetimes by a factor of 3 was predicted in monolayer WSe_2_, suggesting promising applications of ultrathin TMDs in solar energy and electronics^68^ (Fig. 3e). By application of uniaxial strain of 0.2%, a highly tunable nonlinear Hall effect was observed in electron-doped polar TMDs MoSSe thanks to the spin–orbit coupling, providing a potential scheme for building electrically switchable energy-harvesting rectifiers^69^ (Fig. 3f).

At present, photoluminescence (PL) spectroscopy, adsorption spectroscopy, and first-principles calculations have become the mainstream methods to investigate the electronic properties of ultrathin TMDs under strain.^28,50,70,71^ It should be mentioned that the strain-induced electronic structural transition of ultrathin TMDs are significantly dependent on the type of strain, the type of chalcogenide atoms, and the thickness of ultrathin MX_2_ films. In the following statement, we will illuminate the effects of all these factors one by one.

First of all, we discuss the electronic structure of ultrathin TMDs under different types of strain. Despite the fact that both tensile strain and shear strain can induce a semiconductor-to-metal transition in ultrathin TMDs, their physical mechanisms of metallization are quite different. The tensile strain-induced metallization of group-VI TMDs monolayer can be attributed to the overlapping of p_*x*_-orbitals, while the shear strain-induced metallization of group-VI TMDs monolayer results from the transition between d_*z*^2^_ orbitals.^72^

Meanwhile, although similar lattice and electronic structural evolutions can be achieved in ultrathin TMDs through uniaxial and biaxial strain, biaxial strain should be more effective in modifying the lattice and electronic structure of ultrathin TMDs when compare to uniaxial strain.^44,63^ For instance, a band gap transition from *K*–*Σ*_min_ to *Γ*–*Σ*_min_ is expected to occur at a much larger uniaxial compressive strain when compared to the biaxial compression strain.^73^ A semiconductor to metal transition in monolayer MX_2_ can be obtained more easily by application of biaxial tensile strain when compared to the uniaxial strain due to the overlapping of d_*z*^2^_ orbital at Fermi level.^72^ Moreover, a biaxial tensile strain can lead to a much larger increasing carrier mobility in monolayer MoS_2_ when compared to the uniaxial tensile strain.^74^

In addition, the strain direction should also be an important factor which can significantly influence the electronic structural evolution and electrical transport properties of ultrathin MX_2_. For monolayer MoS_2_, a uniaxial strain perpendicular to the transport axis can effectively tune the band gap of monolayer MoS_2_ in a wide range without changing the conductivity significantly, while a parallel strain results in a decreased band gap and a reduced conductance for the electron energies lower than the Fermi energy.^44^ In addition, the mobility of monolayer MoS_2_ is hardly affected by a tensile uniaxial strain along the zigzag direction, while it increases for tensile uniaxial strain along the armchair direction.^74^

Secondly, it should be mentioned that the electronic structure of ultrathin TMDs under strain are significantly sensitive to the type of chalcogens/transition metal atoms. For group-VI TMDs, less shear strain and more tensile strain is required to attain a direct-to-indirect band gap transition in heavier chalcogens owing to its diffuse nature,^72^ and less (more) compressive strain is needed to achieve a semiconductor-to-metal transition in heavier chalcogens (transition-metal) atoms.^75^ At the same strain, monolayer WS_2_ possesses the lightest effective mass among the group-VI TMDs MoS_2_, MoSe_2_, WS_2_, and WSe_2_,^76^ indicating that monolayer WS_2_ should be a more excellent candidate in high performance electronic devices when compared to the rest of the group-VI TMDs.

**Fig. 4:**
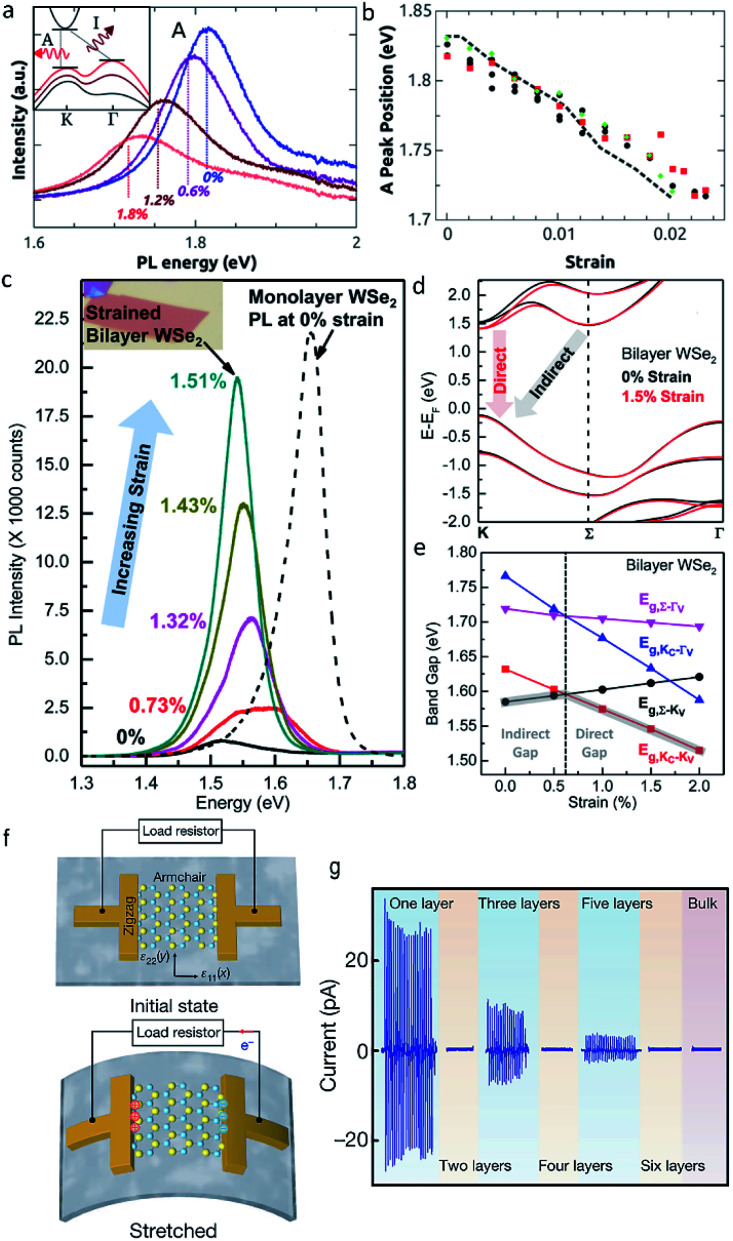
(a) The PL spectra of monolayer MoS_2_ under different tensile strain. (b) Energy of peak A as a function of strain. Reproduced with permission.^50^ Copyright 2013, American Chemical Society. (c) The PL spectra of bilayer WSe_2_ under different tensile strain. (d) Calculated bandgap structure of bilayer WSe_2_ at the strain of 0% and 1.5%. (e) Calculated bandgap energies for different transitions in bilayer WSe_2_ as a function of strain. Reproduced with permission.^80^ Copyright 2014, American Chemical Society. (f) Schematic diagram of monolayer MoS_2_ piezoelectric device. When the device is stretched, piezoelectric polarization charges of opposite polarity are induced at the zigzag edges of monolayer MoS_2_. (g) The piezoelectric outputs as a function of layer number in ultrathin MoS_2_. Reproduced with permission.^82^ Copyright 2014, Nature.

Third, the electronic structure and physical property of strained ultrathin TMDs are strongly dependent on their thickness. Influenced by the interlayer coupling and quantum confinement effect, monolayer and multilayer MX_2_ under tensile strain will exhibit obviously different electronic structures, optical and piezoelectric properties. An application of the uniaxial tensile strain on monolayer MX_2_ (M = Mo, W; X = S, Se) will lead to a redshift of photoluminescence (PL) energy thanks to the narrowed band gap, along with a reduction of the PL intensity at the *K* point of the Brillion zone owing to the transition from direct to indirect band gap.^31,50,77–79^ (Fig. 4a and b). Whereas, tensile strain can result in an enhanced PL intensity in ultrathin (2–4 layers) WS_2_ and WSe_2_ owing to the strain-induced indirect to direct bandgap transition,^77,80,81^ (Fig. 4c–e) which makes it promising in fabricating novel photoelectric devices. In addition, previous experimental study of the piezoelectric properties of ultrathin MoS_2_ confirmed that, stretching and releasing the substrate cyclically can lead to an oscillating piezoelectric voltage and current outputs in MoS_2_ with an odd number of atomic layers, however, this effect disappeared in MoS_2_ with an even number of layers, which can be attributed to the opposite orientations of adjacent atomic layers^82^ (Fig. 4f and g).

It should be mentioned that the strain-induced changes of electronic structure will undoubtedly lead to the electrical transport properties evolution in ultrathin TMDs. When it comes to the electrical transport properties, we have to refer to the “effective carrier mass”. The effective carrier mass can be effectively modified by strain owing to the changes of the covalence of the bond in ultrathin MX_2_ upon strain,^83^ leading to a tunable carrier mobility since the carrier mobility is inversely proportional to the square of carrier effective mass, according to the deformation potential (DP) theory raised by Bardeen and Shockley.^84^ To date, many works have been reported on the simulations about the carrier mobility of ultrathin TMDs under strain. For instance, the electron mobility of monolayer MoS_2_ can be dramatically increased over 10 times with the biaxial strain of 9.5%.^74^ The mobility cut-on rate of anisotropic monolayer ReS_2_ can be enhanced to almost 6 times of the original value with compressive strain of 3%.^85^ All these studies undoubtedly pave the way for a possible application of ultrathin TMDs in electron devices since the external strain can be easily applied in TMDs films by means of specific substrate in fabrication.

The investigations of strain modified mobility of ultrathin TMDs will undoubtedly stimulated researchers to study the effect of strain on the performance of TMD based field-effect transistors. Previous density functional theory suggested that, application of tensile strain can lead to the increase of direct-current performance of monolayer-TMDs-based-double gate field-effect transistors owing to the small energy distance between K- and Q-valleys for the TMDs.^86^

In addition, dipole transition preference of ultrathin TMDs can be investigated from their dielectric properties, thus, the studies on the strain-induced dielectric properties of ultrathin TMDs are of great significant in fabricating nanoelectromechanical devices.^66,87^ According to the previous density functional theory (DFT) calculations, the dielectric properties of ultrathin TMDs are strongly dependent on the type of strain. For monolayer MoX_2_ (X = S, Se, Te), tensile strain showed a stronger displacement towards lower energy in the imaginary part of dielectric function when compared to compression strain, and the application of tensile strain and asymmetric biaxial strain lead to the increase of static dielectric constant, while the application of compression strains result in the first decrease and then increase of static dielectric constant.^43^

At present, the investigations on the electrical transport properties of ultrathin TMDs under strain are still based on the theoretical calculation. First of all, the band structure of ultrathin TMDs under strain is calculated based on the first-principles calculations, subsequently, effective mass (m*) of charge carrier is calculated according to the calculated band structure since the carrier effective mass is directly related to the energy band curvature,^88^ then, the carrier mobility is evaluated based on the linearized Boltzmann transport equation (BTE).^89^ However, it is worth mentioning that the calculated carrier mobility based on the linearized Boltzmann transport equation is imprecise since a number of factors have been ignored during the simulation of the transport processes, such as the neglect of the coherence and interaction among various particles. Thus, realizing the successful measurements of the electrical transport properties of strained ultrathin TMDs is a key issue that remains to be resolved.

**Fig. 5:**
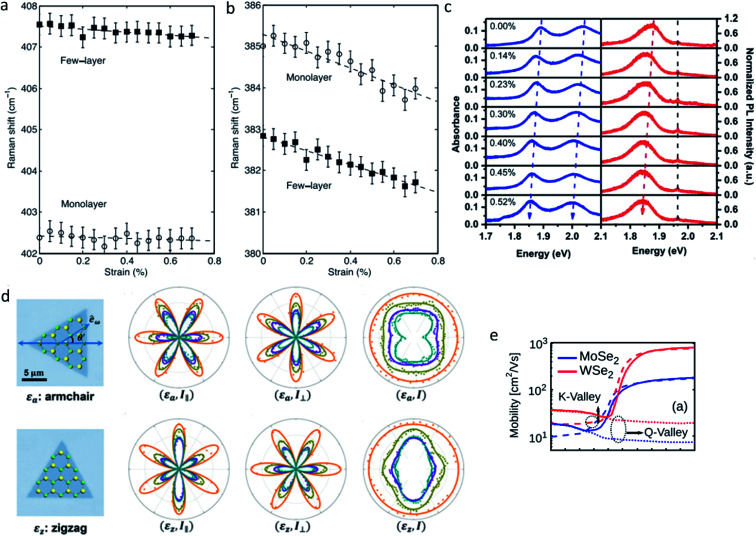
The Raman frequencies of (a) A_1g_ and (b) E^1^_2g_ mode of monolayer and few layer MoS_2_ as a function of strain. Reproduced with permission.^48^ Copyright 2013, American Physical Society. (c) The absorption (left panel) and PL (right panel) spectra of monolayer MoS_2_ under tensile strains. Reproduced with permission.^78^ Copyright 2013, American Chemical Society. (d) Pattern evolution of SHG intensity for monolayer MoSe_2_ under strain, *θ*′ denote the angle between the laser excitation polarization and the strain direction. The strain magnitude is estimated from the SHG intensity, and the strain direction is judged based on the polarization-dependent SHG pattern. Reproduced with permission.^92^ Copyright 2017, American Chemical Society. (e) The phonon limited mobility as a function of strain in monolayer MoSe_2_ and WSe_2_. Reproduced with permission.^93^ Copyright 2015, American Institute of Physics.

The discussion above mainly focuses on the research progress and open issues about the strain-tuned electronic, optical and electrical transport properties of ultrathin TMDs. Inversely, various optical and electrical techniques have also been demonstrated to be efficient and convenient in probing strain in ultrathin TMDs. The strain imaging at submicron scale can be obtained by performing micro-Raman,^48,90^ absorption, photoluminescence spectroscopy,^21,28,78^ since both the Raman frequencies and optical band gap of ultrathin TMDs are sensitive to strain engineering (Fig. 5a–c). Furthermore, the extraction of the full strain tensor with a spatial resolution below the optical diffraction limit can be realized by performing polarization resolved second harmonic generation (SHG) measurements, based on the strain-sensitive nonlinear susceptibility tensor due to a photoelastic effect^91,92^ (Fig. 5d). All these optical techniques are nondestructive, allowing for large-area strain imaging with submicron spatial resolution and are simple to set up. In addition, the accurate measurement of mobility of ultrathin TMDs could also be an effective method to calibrate strain, as previous theoretical calculations predicted, the inter-valley phonon limited mobility of single layer MoSe_2_ and WSe_2_ is very sensitive to strain^93^ (Fig. 5e).

#### Magnetic properties

Magnetic effect is one of the emerging topics in 2D materials due to their potential applications in spin electronics. According to the previous experimental and theoretical researches, magnetism can be induced in a nonmagnetic nanomaterial by embedding transition metal (TM) atom due to the partially filled d character of the transition metal atoms,^94–97^ thus, it is expected that TMDs with a particular d character may contain a range of magnetic behaviors. However, this is not the case, most of the TMDs are nonmagnetic in themselves at ambient condition, which can be attributed to the fact that, the TM atoms M in TMDs are sandwiched between two layers of chalcogens atoms X, forming a strong ligand field due to the M–X covalent bond and thus quenching the magnetism of M atoms. Strain engineering should be a useful method to reduce the covalent interactions between M–X bonds, in this way, investigating the magnetic properties of strained TMDs should be of great significant in exploring the spintronics in 2D materials.

In the past decades, substantial researches about the magnetic properties of strained ultrathin TMDs have been reported, which mainly focus on the theoretical prediction of magnetic moment, magnetism order and strength of magnetic coupling under strain. Intriguing magnetization behaviors and magnetic transition have been obtained in strained ultrathin TMDs owing to their strain-induced lattice and electronic structural evolution.^98–100^

Despite the fact that the vast majority of TMDs are nonmagnetic at ambient condition, there also exist very rare magnetic TMDs, such as group-V TMDs VX_2_ (X = S, Se). It should be mentioned that their strain-induced magnetic property evolutions are strongly dependent on the types of strain. For instance, previous density functional theory suggested that, application of in-plane biaxial strain can induce a phase transition of monolayer VS_2_ from semiconducting-H phase to metallic-T phase, accompanied by a high spin to low spin magnetic transition.^99^ While application of isotropic strain can lead to increasing magnetic moments and strength of magnetic coupling in VS_2_ and VSe_2_ monolayers, the increasing magnetic moments can be attributed to the increasing bond length of d_v–x_, namely the increasing ionic composition of the interaction between the V and S/Se atoms, while the increasing magnetic coupling arise from the combined effects of both through-bond and through-space interactions.^100^

It is worth mentioning that, for intrinsically nonmagnetic ultrathin TMDs, their strain-induced magnetism are depending on not only the increasing bond lengths with strain but also the particular metallic character of ultrathin TMDs.^98^ That is, magnetism behaviors can be observed in strained metallic TMDs, such as group-V TMDs MX_2_ (M = Nb, Ta; X = S, Se), while the strained semi-conductive TMDs such as group-VI and group-X TMDs MX_2_ (M = Mo, W, Pt; X = S, Se) cannot be magnetized thanks to the presence of certain band gap.^98,101–103^ Actually, similar situations have also been observed in other 2D materials, such as Ni-substituted graphene and nanotubes where only metallic structure can be magnetized under strain.^95,96^

Despite the fact that strain engineering alone cannot induce magnetism in semi-conductive TMDs, combination of strain engineering with other modifications, such as doping and hydrogenation, is an effective technique to introduce and manipulate the magnetism in ultrathin group-VI TMDs thanks to the localizing unpaired 3d electrons of TM atoms and the controllable strength of the spin-splitting of TM-3d orbitals under strain. Next, the magnetic behaviors of strained semi-conductive TMDs are discussed from two aspects: doping and hydrogenation.

**Fig. 6:**
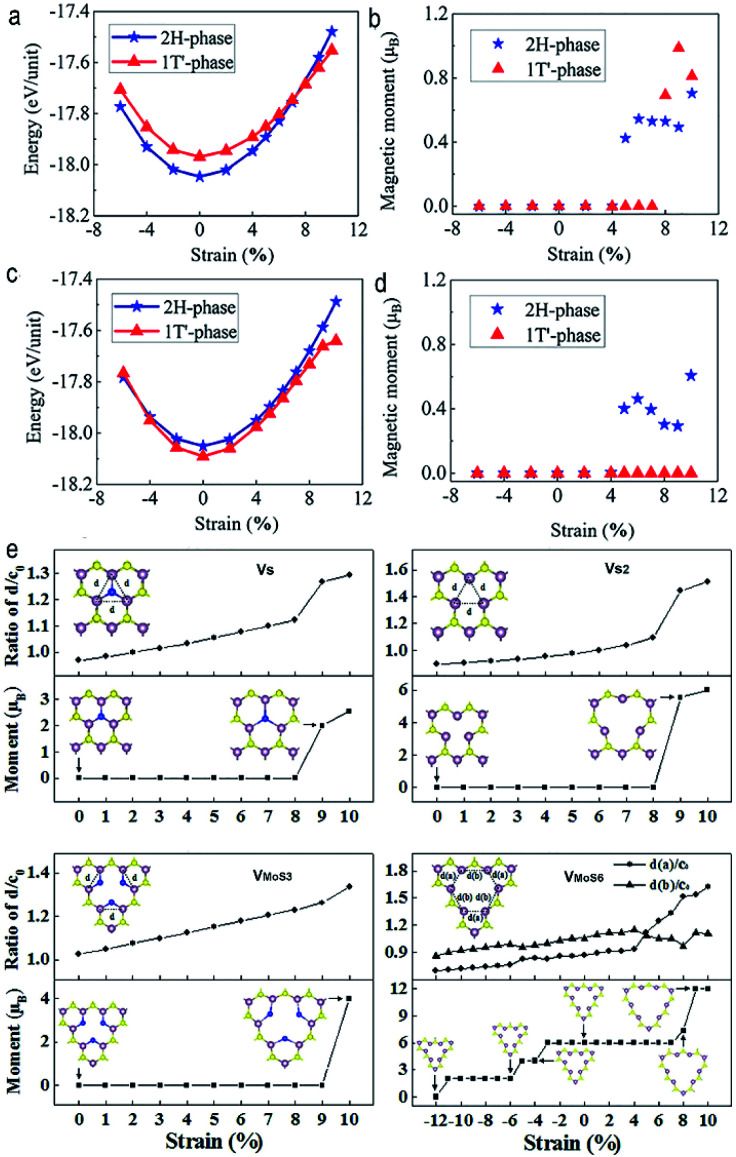
(a) Total energy and (b) magnetic moment of Mo_0.9_Re_0.1_Te_2_ as a function of tensile strain. (c) Total energy and (d) magnetic moment of Mo_0.875_Re_0.125_Te_2_ under different strain. Reproduced with permission.^104^ Copyright 2020, American Chemical Society. (e) Strain dependent magnetic moments for the V_S_, V_S2_, V_MoS3_, and V_MoS6_ doped monolayer MoS_2_. Reproduced with permission.^106^ Copyright 2014, American Institute of Physics.

On the one hand, the “doping” here refers to the TM atoms embedding or the vacancies in ultrathin TMDs. Application of tensile strain can lead to magnetism in TM-atom-doped TMDs films, even magnetic transformation in TM atoms substituted TMDs, which can be attributed to the conversion from covalent to ionic bonding between TM atoms and chalcogens atoms.^104,105^ For instance, by application of tensile strain of 8%, a strain-induced phase transition from 2H phase to 1T phase can be obtained in the relatively low Re doping concentration in monolayer MoTe_2_, accompanied by a sudden magnetization phenomenon (Fig. 6a–d). In addition, the combination of vacancy doping and strain engineering can also lead to magnetism in ultrathin TMDs. Previous first-principles calculations suggested that, under tensile strain, magnetism can be introduced and manipulated in vacancies doped monolayer MoS_2_ owing to the breaking or the change of Mo–Mo metallic bonds around the vacancies^106^ (Fig. 6e).

**Fig. 7:**
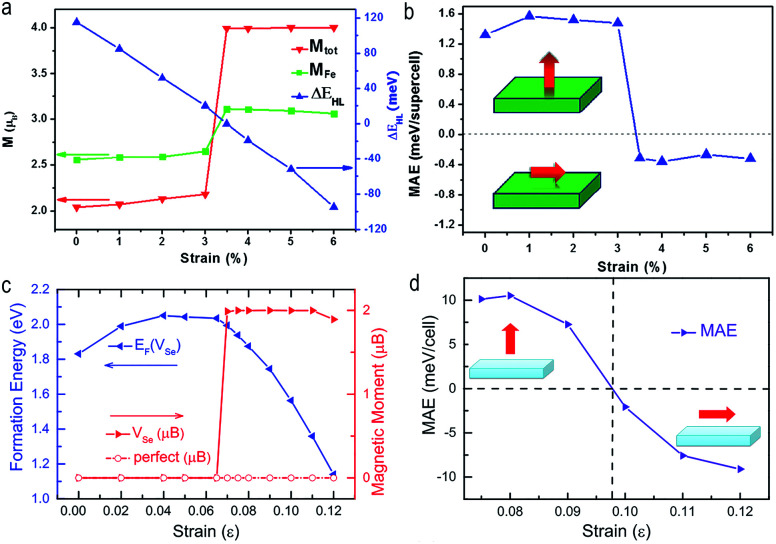
(a) Magnetic moment (vertical coordinate on the left) and the energy difference between high spin and low spin (vertical coordinate on the right) of Fe doped monolayer MoS_2_ as a function of strain. (b) The strain-dependent magnetocrystalline anisotropy energy (MAE) in Fe doped monolayer MoS_2_. Spin reorientation transition from out-of-plane to in-plane magnetization occurs when the strain is larger than 3%. Reproduced with permission.^107^ Copyright 2015, Elsevier. The strain-dependent (c) formation energy of V_Se_ and total magnetic moments of V_se_ doped monolayer PtSe_2_. (d) MAE in V_se_ dopped monolayer PtSe_2_. The strain induced magnetization direction transition occurs in V_se_ dopped monolayer PtSe_2_ at the strain of 0.1%. Reproduced with permission.^103^ Copyright 2016, American Institute of Physics.

Moreover, not only the magnetism, but also the magnetization direction of ultrathin TMDs can be effectively modified by combination of doping and strain engineering. A magnetic state transition from low spin to high spin and a spin reorientation transition from out-of-plane to in-plane magnetization were predicted in strained Fe-doped monolayer MoS_2_.^107^ Similar situation were also demonstrated in strained monolayer PtSe_2_ with Se vacancy^103^ (Fig. 7).

On the other hand, using atomic hydrogen to functionalize the nonmagnetic TMDs can not only form a chemical bond easily with the chalcogens atom but also serve as an electron donor to the host, in this way, the perfect combination of hydrogenation and strain engineering will provide a great opportunity for the introduction and modification of magnetic properties in ultrathin TMDs. For instance, a strain-induced ground-state transition from nonmagnetism to ferromagnetism (FM) accompanied with enhanced magnetic moment and stability was predicted in hydrogenated MoS_2_ and hydrogenated MoSe_2_.^108^

At present, the magnetic properties studies of ultrathin TMDs are mainly based on the theoretical calculations, the relative experimental evidences are still scarce due to the restriction of the experimental facility. When it comes to the relatively fewer experimental observations, it should be mentioned that magnetic force microscopy (MFM) is an effective experimental facility which can be used to explore the strain-induced magnetization behaviors since it can distinguish the magnetic and nonmagnetic responses at nanometer scale.^109^ with the aid of MFM, Yang *et al.* have investigated the strain-induced magnetic property of monolayer ReSe_2_, where local strain was introduced on top of the wrinkled monolayer ReSe_2_. Magnetic phenomenon was observed at the wrinkle regions in monolayer ReSe_2_, evidenced by the reverse shift of the MFM phase and amplitude images. Further density functional theory also demonstrated a more stable magnetic state of strained monolayer ReSe_2_ when compared with the nonmagnetic states.^70^

It should be mentioned that, sometimes, only using theoretical calculation method to predict the magnetic behaviors of strained TMDs films may lead to controversial conclusions. For instance, Zhou *et al.* predicted a tensile-strain-induced magnetization with a ferromagnetic character in monolayer NbS_2_ and NbSe_2_.^98^ While Xu *et al.* predicted a tensile-strain-induced transition from antiferro- to ferro-magnetism in both monolayer NbS_2_ and NbSe_2_.^110^ It is obvious that this contradiction cannot be solved by just relying on the theoretical calculations, more experimental evidences are required to solve this controversy. Thus, it is time to develop the relative experimental techniques to provide more intuitive results and promote the progress of studies on the strain-induced magnetic properties of ultrathin TMDs.

## Challenges and opportunities

According to the previous theoretical reports, most of the TMDs films are predicted to be capable of sustaining much larger strain when compared with their bulk counterparts, however, microcracks may appear in TMDs-based devices even at small strain, which can be attributed to the defects and the brittleness in ultrathin TMDs.^111,112^ Since the existence of defects, such as doping, vacancies, and grain boundaries, are inevitable during the preparation of TMDs films, it is still a great challenge to fabricate ultrathin-TMDs-based devices capable of surviving large strain.

In addition, an immense amount of theoretical researches have been reported during the past decades, however, the development of experimental researches always lag behide the theoretical researches. Firstly, despite great progress has been made in the synthesis of large-area uniform TMDs films,^11^ the restriction of facility remains a significant problem in realizing effective modification of the thermal transport, electrical transport, and magnetic properties in strained ultrathin TMDs. Secondly, although the difference of strain-induced physical properties along the armchair and zigzag directions is small for isotropic ultrathin TMDs, there is a gap between theoretical and experimental studies on the uniaxial strain in anisotropic TMDs films. In detail, for most of the theoretical calculations and simulations, the strain effects of anisotropic TMDs films are strictly separated according to the strain directions, while when it comes to the experimental operation, the precise control of strain engineering along the armchair and zigzag direction is still difficult due to the huge challenges in exact crystallographic strain actuation and measurements. To confirm the existing prediction and reduce the distance between theory and experiment, it is time to accelerate the development of experimental technology.

## Conclusions

In this review, we first focus on the experimental acheivements in approaches for introducing uniaxial, biaxial, and isotropic strain in ultrathin TMDs, the characteristics and merits of each approach have been summarized. Subsequently, the research progress in the modification of physical properties in strained ultrathin TMDs, including both experiment observations and theoretical calculations, were outlined by categorizing the physical properties: (1) lattice structure, phonon properties and thermal transport properties; (2) electronic, optical and electrical transport properties; and (3) magnetic properties. Futuristically, fabricating ultrathin-TMDs-based devices capable of surviving large strain, and trying hard to explore the experimental facilities and conditions to reduce the distance from theoretical calculations, should be the only way to achieve great success in designing TMDs based devices.

## Conflicts of interest

There are no conflicts to declare.

## Supplementary Material
